# Region of Interest analysis using mass spectrometry imaging of mitochondrial and sarcomeric proteins in acute cardiac infarction tissue

**DOI:** 10.1038/s41598-018-25817-7

**Published:** 2018-05-10

**Authors:** Yuka Yajima, Takuya Hiratsuka, Yu Kakimoto, Shuichiro Ogawa, Keisuke Shima, Yuzo Yamazaki, Kenichi Yoshikawa, Keiji Tamaki, Tatsuaki Tsuruyama

**Affiliations:** 1 Department of Microbiology, Muroran Institute of Technology, Muroran, Hokkaido 050-8585 Japan; 2 Department of Drug and Discovery Medicine, Pathology Division, Kyoto University Graduate School of Medicine, Kyoto, 606-8501 Japan; 3 Department of Forensic Medicine, Graduate School of Medicine, Tokai University School of Medicine, Isehara-Shimokasuya 143, Kanagawa, 259-1193 Japan; 4 Center for Anatomical, Pathological, and Forensic Medical Research, Kyoto University Graduate School of Medicine, Kyoto, 606-8501 Japan; 5 Kyoto Applications Development Center, Analytical & Measuring Instruments Division, Shimadzu Corporation, 1 Nishino-kyo-Kuwabara-cho, Kyoto, 604-8511 Japan; 6 Department of Life and Medical Sciences, Doshisha University, 1-3 Tatara Miyakodani, Kyotanabe-shi, Kyoto, 610-0394 Japan; 7 Department of Forensic Medicine, Kyoto University Graduate School of Medicine, Kyoto, 606-8501 Japan

## Abstract

Matrix-assisted laser desorption ionization image mass spectrometry (MALDI-IMS) has been developed for the identification of peptides in various tissues. The MALDI-IMS signal distribution patterns and quantification of the signal intensities of the regions of interest (ROI) with healthy regions were compared for identification of the disease specific biomarkers. We performed a new ROI analysis using the conventional *t-*test and data number independent Cohen’s *d*-value analysis. Using these techniques, we analysed heart tissues after acute myocardial infarction (AMI). As a result, IMS signals of mitochondrial adenosine triphosphate synthase alpha subunit (ATP5A), myosin-6/7(MYH6/7), aortic actin, and the myosin light chain 3 (MYL3) were identified in the infarcted region. In particular, the signals of MYH7 are significantly greater in the infarcted region using ROI analysis. ROI analysis using MALDI-IMS may be a promising technique for the identification of biomarkers for pathological studies that involve the comparison of diseased and control areas.

## Introduction

Imaging mass spectrometry (IMS) is a technique used to visualize the spatial distribution of chemical compositions by their *m/z* of molecular masses (*m*) to electron charge (*z*) and measurement of (phospho) lipids and other low weight molecular drugs^1–3^. Over the past several decades, secondary ion mass spectrometry (SIMS) imaging mass spectrometry (SIMS-IMS) has played a pivotal role in pharmacological monitoring. On the other hand, Matrix-assisted laser desorption ionization (MALDI) imaging mass spectrometry (MALDI-IMS) technique was developed for the analysis of relatively large molecules. It has recently been shown that MALDI-IMS is the most effective IMS for analysis of tissue. In this technique a tissue section is utilized on the slide glass while the mass spectrum is recorded. Pharmaco- and toxico-dynamics have been studied broadly by MALDI-IMS. Although MALDI-IMS is used for the discovery of biomarkers for clinical diagnosis, further development is required for the measurement of peptides and proteins^4–14^, as there are limitations in the quantitative applications of two-dimensional (2D) imaging and correlation analysis of histopathologic diagnostic data. To overcome the difficulties in analysis of 2D based MALDI-IMS, we recently developed a novel IMS program, Imaging MS Solution® for region of interest (ROI) analysis. Intensity value information for all pixels in each *m/z* image was treated as one dataset in a mathematical data-matrix (MDM). The intensity value of each pixel is summarized, in order, in one column for the *m/z* of each spectrum in MDM. This ROI analysis is expected to assist comparison of substances in two specified regions of interest (disease lesion areas vs. health intact areas) to identify substances that are increased or decreased in each region.

As the objective of our study, we selected acute cardiac infarction tissue. In a previous study, we identified a promising biomarker for myocardial infraction, SORBS2^15^, observed in the Z-band and intercalated disk^16^, using liquid chromatography (LC)/MS techniques. In this current study, we tried to image infarcted lesions in the heart tissue as an ROI by identifying mitochondrial and sarcomeric proteins that would reflect cardiomyocyte viability. Histological analysis is one of the key methods that allows for studies on cardiac muscle damage; however, the lesions observed are not sufficiently informative about the subsequent phase that immediately follows the onset of the infarction episode^17^. Wavy fibres and contraction bands, which are histological hallmarks of acute myocardial injury^18^, are localised and not always obvious. Thus, the earliest histological change is rarely visible with the onset of AMI, and retrospectively evaluations of the range of infarction, using routine histological tools, remains difficult. Here, we anticipated that ROI analysis using MALDI-IMS would enable us to determine the range of cardiac damage, thereby demonstrating the potential of this technique to aid in histological diagnoses. Therefore, histologic proteomics studies, based on ROI analysis using MALDI-IMS, represent a promising approach for discovering novel diagnostic markers and understanding the pathogenesis of cardiac remodelling^19^.

## Results

### IMS analyses

Initially, to achieve precise histological evaluation using MALDI-IMS^15^, we selected a representative Japanese patient from a pool of patients using the following criteria: (i) death within 7 h of the onset of symptoms, without any angioplasty or thrombolytic therapy, (ii) serum that was positive for heart-type fatty acid-binding protein (H-FABP), as determined using a commercial ELISA kit (Rapicheck^®^; DS Pharma Biomedical Co., Osaka, Japan),^20^ and (iii) observation of contraction bands or wavy fibres in the free wall of the left ventricle (LV) upon histopathology^6,15,21^.

**Figure 1:**
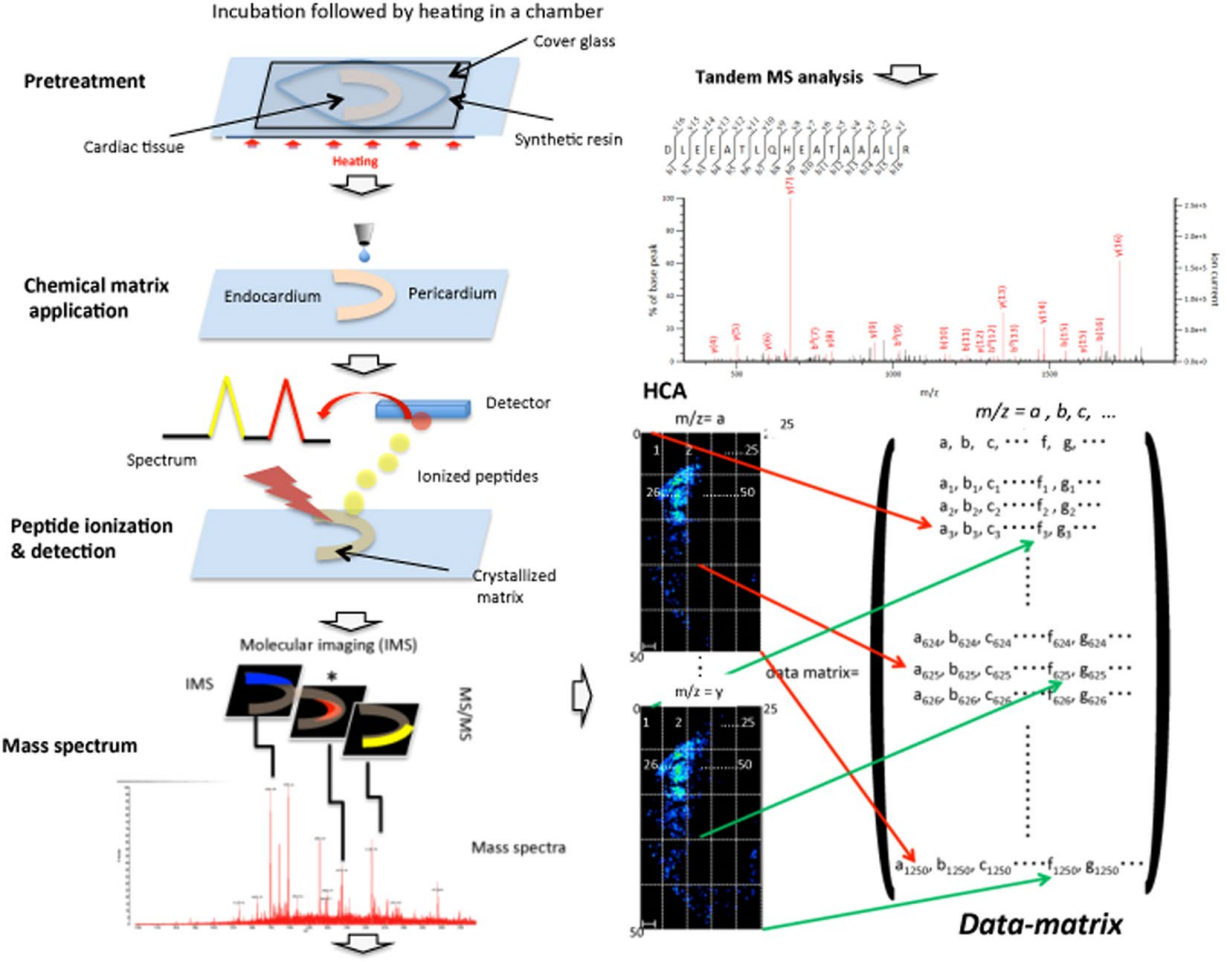
MALDI-Imaging mass spectrometry: experimental flowchart. Cardiac tissue samples were digested using trypsin in a chamber at 37 °C, and then were boiled at 95 °C in a reaction buffer developed in our lab. Following matrix (2,5-dihydroxybenzoic acid) deposition, a laser was used to ionize the peptides and an electric field was applied until they reached the detector. The ionized peptides were separated based on mass-to-charge ratio (*m/z*) values and the spectrum revealed the two-dimensional distribution of the peptides in tissues. Mass spectra were generated in an ordered array in the x-y coordinate space. Individual spectral features were visualised within the tissue to generate protein images. Histological features within the sample were correlated with molecular species. MS/MS analysis identified proteins that corresponded to individual spectra. For example, the lateral and axial resolution of an image is 25 × 50 pixels. The intensity of every pixel is measured for each *m/z* value. The MDM obtained from this dataset is shown in the right bottom panel. In this MDM, the row represents individual *m/z* values (*a, b, c…*); the columns indicate the *m/z* peak values of all 1250 pixels (*m/z*_*j*_;1≤ *j* ≤ 1250).

FFPE tissues were cut into sections with a 3–4 μm thickness and firmly mounted on indium tin oxide-coated (ITO) glass. Samples 1–3 were obtained from the patient. Samples 1 and 2 included tissues with an infarcted area containing contraction bands, dilated arterioles, and endothelial damage in the endocardial region. Sample 1 had severe infarction in the endocardium, showing wavy fibres, while sample 2 showed mild infarction spanning the entire tissue. Sample 3 did not contain an infarcted area. Samples were pre-treated using a method that we had previously developed (see methods) and subsequently analysed using MALDI-IMS (Fig. 1).

**Figure 2:**
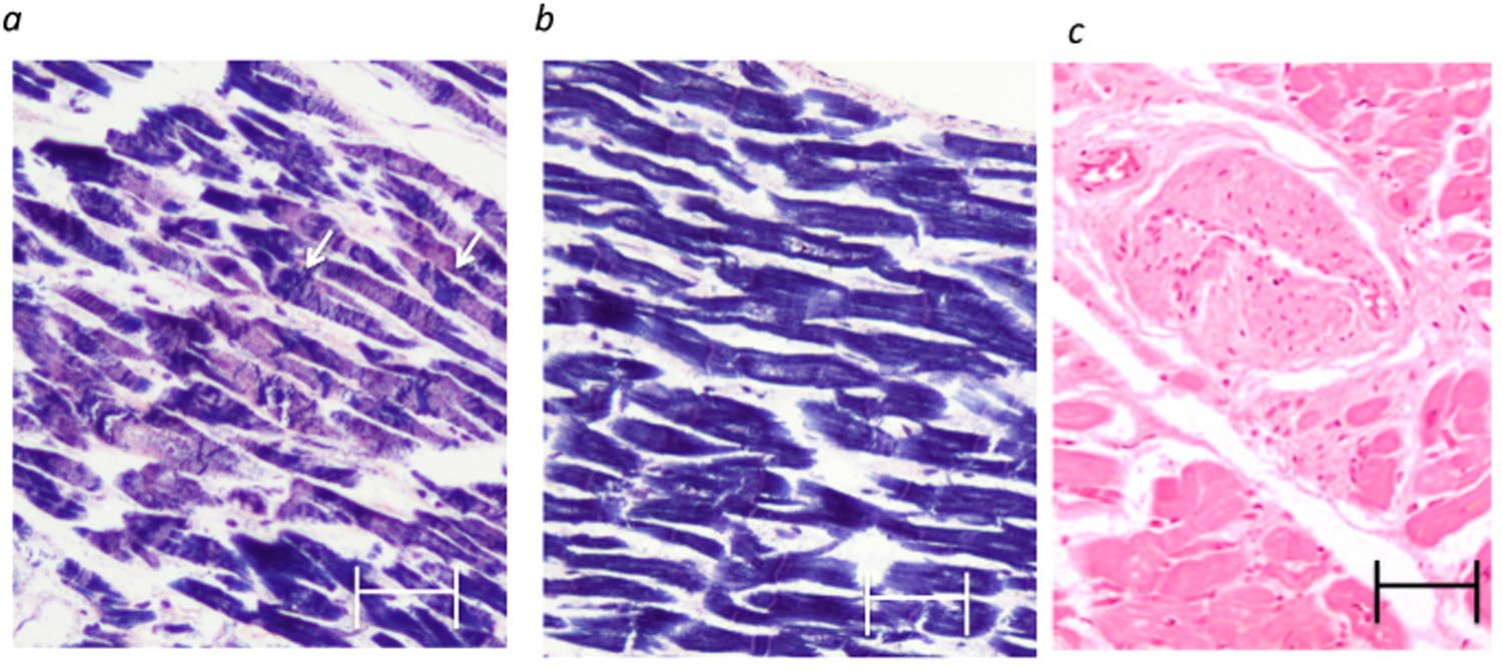
Histological analyses of samples. Representative PTAH staining (**a**,**b**) and H&E staining of the contraction bands in the ischemic endocardial lesion. Arrowheads, contraction bands (**a**). Damaged arterioles with intimal thickening (**c**). Scale bars, 20 μm in (**a**–**c**).

After staining with haematoxylin and eosin (H&E) or phosphotungstic acid-haematoxylin (PTAH), two samples of infarcted cardiac tissue were found to contain damaged cardiomyocytes, revealing contraction bands in the LV (Fig. 2a; intact myocytes are shown in Fig. 2b) and arterial occlusion in the area of endocardial papillary muscles (Fig. 2c).

### MALDI-IMS and tandem MS analyses

Using the cardiac tissues, we obtained MALDI-IMS signals that corresponded to a total of ~50–60 peptides. IMS profiles of the cardiac tissue samples and representative data of the proteins identified by IMS are summarized in Supplementary Table 1. IMS signals of haemoglobin subunit α (HBA) were used to evaluate the amount of blood oxygen supply (as HBA is a carrier of oxygen supplies for cardiomyocytes) and, accordingly, the diagnosis of ischemic or infarcted areas. To confirm the MALDI-IMS data, heart tissues were directly subjected to LC/MS and all proteins were detected whose fragments were identified by in-tissue MS/MS (Supplementary Table 2; Supplementary Fig. 1a–c).

**Figure 3:**
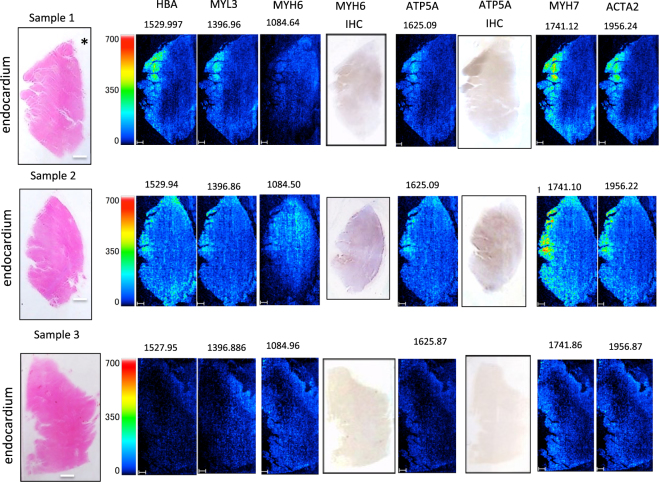
MALDI-IMS analyses. HBA, ATP5A, MYH6, MYH7, MYL3, and ACTA2 IMS signal analyses (for samples 1, 2, and 3) were obtained using MALDI-TOF-MS. The ions were identified using the same tissue samples as in the MS/MS experiments. Scale bars, 500 μm. H&E staining images and orientation (endocardium and pericardium) along with ROI are indicated in the left column. The identified proteins are indicated at the top; *m/z* values are shown above the images. IHC represents immunohistochemistry of individual proteins. Asterisks “*” represent the coronary artery.

**Figure 4:**
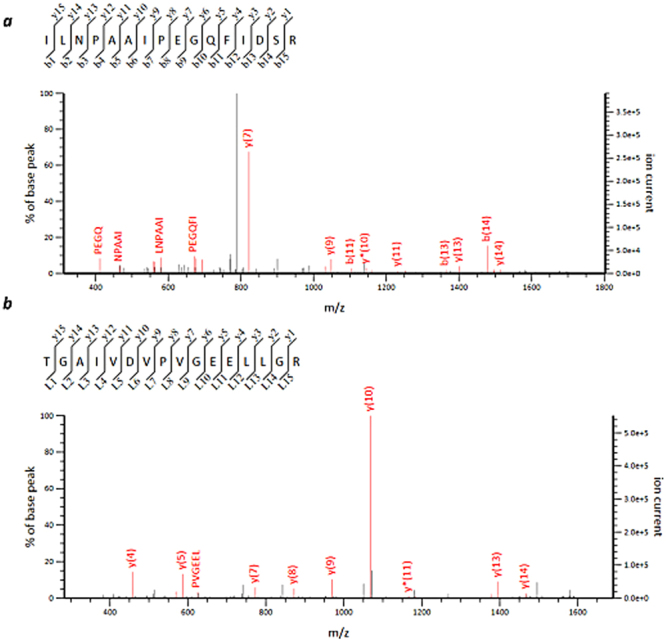
Tandem MS (MS/MS). Tandem mass spectrum of the precursor ion in the infarcted cardiac tissue (sample 1) at (**a**) HBA, *m/z* 1529, *P* = 2.6 × 10^−5^; (**b**) ATP5A, *m/z* 1625, *P* = 6.2 × 10^−5^; and (**c**) MYL3, *m/z* 1396, *P* = 1.2 × 10^−2^.

We then examined whether HBA was localised around the coronary artery in samples 2 and 3 (Fig. 3 in the left column) on the pericardial side of the tissue, as expected, to investigate any correlation between the intensity of the HBA signal and amount of blood flow. In contrast to our expectations, no HBA signal was detected on the pericardial side of the coronary artery occlusion in sample 1 (Fig. 3), whereas the signal was more intense in the endocardium, suggesting either haemodynamic abnormality or cardiac tissue damage arising from haemorrhagic ischemia. Tandem MS/MS was subsequently used to analyse the peptides that corresponded to the *m/z* values, and 1529.8 (HBA) was identified (Fig. 4, Supplementary Table 1) in MALDI-IMS from samples 1–3. Subsequently, proteins with a *m/z* of 1084 (myosin heavy chain 6: MYH6), 1396 (myosin light chain 3: MYL3), 1625 (ATP5A), 1741 (myosin heavy chain 7: MYH7), and 1956 (alpha actin 2: ACTA2) were identified using tandem MS/MS (Figs 3, 4a,b, and Supplementary Fig. 2). Images from the immunohistochemical analyses of ATP5A and MYH6 are shown in Fig. 3. Staining signal of ATP5A was not evident in the intact pericardial area (right side of the tissue image shown in Fig. 3) in sample 3. By contrast, ATP5A staining was intensely observed in the infarcted muscle fibres of the subendocardial area with contracted myocytes (left side of the tissue image in Fig. 3 in samples 1 and 2. Overall, higher ATP5A signal intensities were observed in the infarcted area. Similarly, staining of MYH6 was intensely observed in papillary muscle subendocardial lesions by ATP5A immunostaining (samples 1 and 2), which was in accord with the MALDI-IMS data. By contrast, normal sample 3 did not exhibit definitive immunohistochemical staining for either of the antibodies; these findings were also consistent with the MALDI-IMS data.

### ROI analysis based on MALDI-IMS

**Figure 5:**
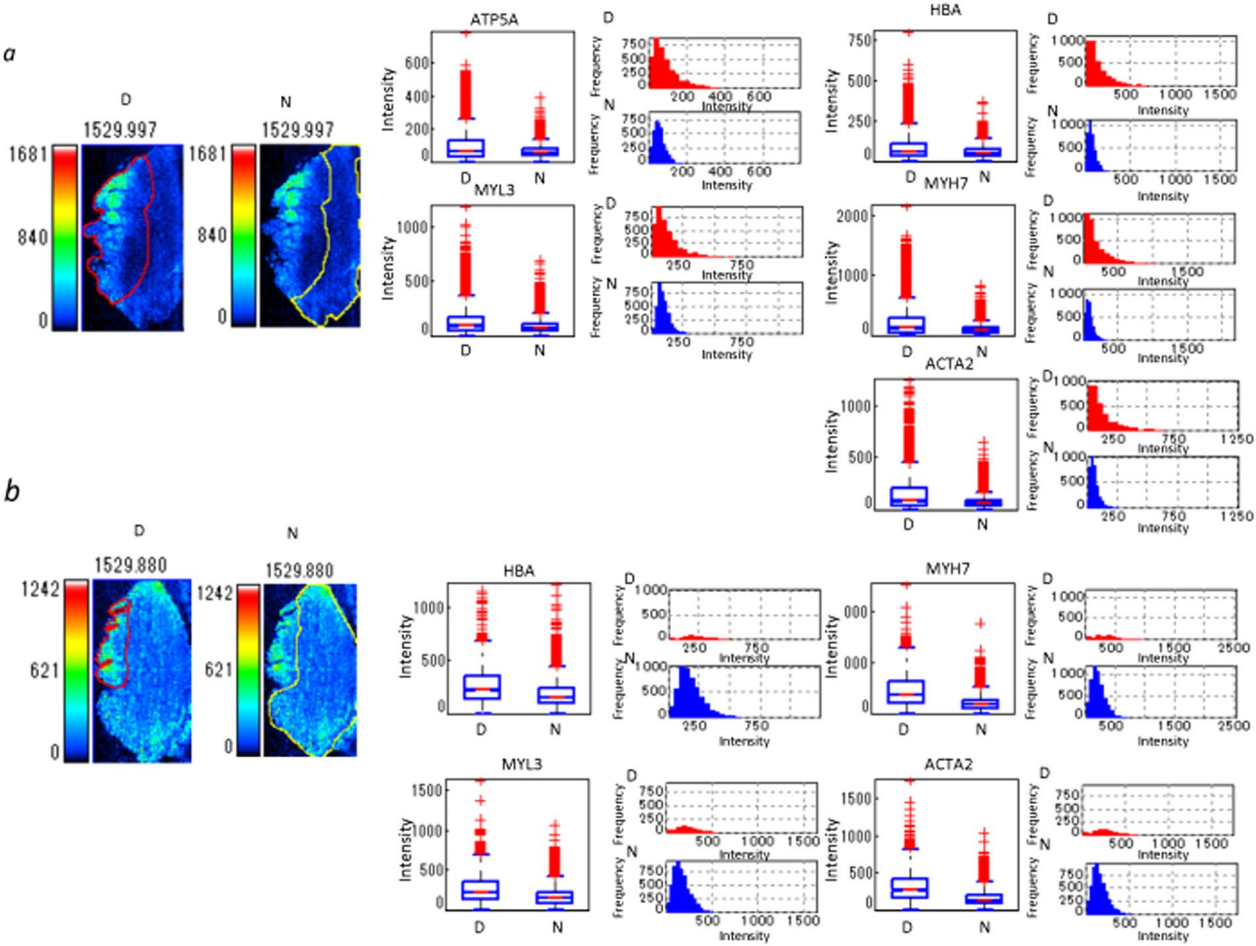
Quantification of peptides in infarcted and healthy areas by ROI analysis. The box plot shows differences and the distribution of intensity of all pixels in a *m/z* image between infarcted and healthy area. Box plot: upper horizontal line of box, 75^th^ percentile; lower horizontal line of box, 25^th^ percentile; horizontal bar within box, median; upper horizontal bar outside box, 90^th^ percentile; lower horizontal bar outside box, 10^th^ percentile. The histogram shows differences and the frequency of intensity of all pixels of the *m/z* image between infarcted and healthy areas. The histogram vertical axes represent the frequency of pixels. The horizontal axes represent intensity. (**a**) Sample 1, (**b**) sample 2. The two photos on the left represent individual ROIs. The red lines demarcate the border of the ROI for the endocardial infarcted lesion (***D***) in the left side of the tissues and of the residual normal area (***N***) in the right side of intact area. The yellow lines demarcate the ROI of the healthy lesion.

To precisely quantify the HBA signal, the average signal intensities in the infarcted and healthy regions were calculated by ROI analysis using Imaging MS Solution®. Based on PTAH and H&E staining, the subendocardial area, including wavy fibres and contraction bands that suggested infarction injury (Fig. 2a), were selected as the ROI from the resting or normal histological area (Fig. 2b). An infarcted ROI was selected based on the observation of contraction bands and endothelial damage (Fig. 5).

The intact region was diagnosed as a region in which the percentage of contraction bands and other myocyte damage was less than 5% at 400 × magnification. HBA signals in the infarcted ROI of samples 1 and 2 were measured for all pixels in the respective regions (Sample 1 − infarction area: *N* = 4126, healthy area: *N* = 7554; Sample 2 − infarction area: *N* = 1054, healthy area: *N* = 8176); statistics are shown in Table 1. We found that the average observed intensity was significantly higher in the healthy regions (Fig. 5a,b; Table 1). Furthermore, mean signal intensities for MYH6, MYL3, ATP5A, MYH7, and ACTA2 were also obtained.

**Table 1:** Comparison of ROI analysis between the infarcted and healthy regions of the sample.

*Sample 1 m/z*	infarction	SD	healthy	SD	*p* value	*d* value	medium
1529.997	179.1	189.9	91.2	59.9	1.88E-171	0.63	medium
1625.124	102	91.5	67.6	38.2	7.88E-109	0.49	small
1741.224	234.5	258	120.1	87.4	5.32E-156	0.6	significant
1396.987	147	141.4	99	65.7	2.09E-86	0.44	small
1956.34	167	187.2	82.9	61.4	8.51E-161	0.61	significant
***Sample 2 m/z***	**infarction**	**SD**	**healthy**	**SD**	*p* **value**	*d* **value**	
1529.88	276.3	192.6	194.7	129.4	2.48E-65	0.57	medium
1741.099	475.5	354	226.8	150	4.09E-284	1.25	significant
1396.839	279.8	194.6	180.2	111.6	1.21E-114	0.77	medium
1956.208	327.7	232	167	110.5	1.21E-247	1.15	significant

The signal intensity of all five proteins described above was increased in the ROI (infarction area) of sample 1 compared to those in the healthy area (Fig. 5a; Table 1, *P* < 0.01). ATP5A signal was not clear in sample 2, while those of the other four proteins were consistently higher in the ROI than in the healthy area (Fig. 5b; Table 1, *P* < 0.01).

In addition, we performed a statistical analysis of ROI using Cohen’s *d*-value^22^ that is independent of data numbers (Table 1). The estimation of the *d*-value was performed via comparison of the data of the endocardium and the pericardium in healthy tissue No. 3 (Supplementary Fig. 3), in which all of the values for peptides were less than 0.8 (Supplementary Table 3). The results revealed that the signal intensity of MYH7 was commonly greater than that in the healthy region in both samples No. 1 and No. 2.

## Discussion

Quantitative comparisons of the distribution of peptide signals in the tissue have been considered to be difficult in MALDI-IMS because of the challenge of computing based on an algorithm using *m/z* for numerous pixels. In addition, big image data compression is critical for prevention of loss of data that was obtained by MALDI-IMS analysis. Therefore, creating MDM based on MALDI-IMS was necessary for ROI analysis of intensity differences between the infarcted and healthy areas in each batch of imaging data for all pixels that was summarized to prevent the subjective and arbitrary selection of measurement points. Performing analyses with a separate ROI, specified for infarcted and healthy regions within the same cardiac tissue, identified molecules that were specific to the infarcted region according to histopathologic evaluation, which could represent potential biomarkers of infarcted tissues.

In analysis of big imaging data, conventional statistics using *t-*test is not necessarily sufficient because *p-*value is inevitably smaller in analysis of a number of data. This is one of the critical issues on the imaging data analysis that is inevitably a set of big data. On the other hand, Cohen’s *d-*value is independent of the numbers of data (refer to methods), which supports *t-*test analysis. By application of this Cohen’s methodology, we could obtain more reliable data of the two dimensional distribution of the signal intensity on the cardiac tissue and found that MYH7 signal was significantly enhanced in the infarction area.

For validation of the current data processing technique, an analysis of whole cardiac tissue is critical in order to analyse cardiac infarction area *in situ*. Many studies have used infarction models to gain a deeper understanding of cardiac muscular damage^23,24^. Cardiac myocytes in the subendocardium, having the minimum blood supply, are very susceptible to infarction damage. Accordingly, acute ischemia primarily affects the subendocardial papillary muscle region, which may affect mitral regurgitation^25^ and lead to reduced myocyte viability. At the beginning of this current study, we hypothesized that a reduction in cardiomyocyte-related proteins in infarct lesions occurred because of leakage of these proteins from myocytes. our observations in MALDI-IMS and immunohistochemical studies suggested enhanced HBA, ATP5A, and sarcomeric protein in the subendocardial damaged lesion. By contrast, the enhanced signal of HBA in the subendocardial papillary muscle suggested aberrant haemodynamics followed by fragmentation into subunits of haemoglobin from the damaged vessels. A previous experimental study showed that the relative resistances in bloods in the subendocarium were higher than that in the subepicardium^26^ and that acute infarction may promote erythrocyte injury by resistance in the circulatory blood. In such cases, fragmented HBA from erythrocytes may become identifiable in the injured myocytes. Clinically, ischemic rupture of the anterolateral papillary muscle has been frequently reported^27^.

Among the identified proteins, ATP synthase, on the inner mitochondrial membrane, catalyses the oxidative phosphorylation during the respiratory process; dysfunction of this step critically affects cardiac performance, hence contributing to heart failure^28–32^. A close relationship between ATP synthase levels and left ventricular mass in patients with ischemic cardiomyopathy was described^33^. The F_0_ complex contains trans-membrane subunits harbours the catalytic sites, providing energy for ATP synthesis and subunits α and β are the core components involved in the physiological cardiac function^31^. As the infarction was acute and of short duration, we did not expect any up-regulation of ATAP5A. Therefore, we hypothesized that ATP5A was exposed at the surface of myocyte due to the ischemic damage, allowing its increased ionization (by its dissociation into subunits) for MALDI-IMS analysis^34–37^. Further, at the early phase of AMI, the detection relapsed and released ATP5A may be detected in the peripheral blood such as creatine kinase, M type^38^. We hypothesize that this myocyte damage in the subendocardial area was accompanied by probable release of other sarcomeric proteins, MYH6, MYH7, MYL3, and ACTA2.

We showed that MYH7 distributions differed between cardiac infarction samples and healthy tissues, suggesting their different involvement in the pathogenesis of cardiac infarction (Fig. 3). In this current study, the signals of both MYH6 and MYH7 were increased in the infarcted cardiac tissue samples compared to those in the intact tissue, as was shown in a mitochondrial proteomics study using a rat model^39^. Recently, several studies reported that MYH7 is associated with cardiac infarction and the development of cardiomyopathy. An opioid receptor agonist that induces persistent protection against ischemic reperfusion (I/R) injury in the heart was shown to induce increased MYH7 levels^40^. ACTA2 represents a vascular smooth muscle cell-specific isoform of α-actin. Alterations in its signal intensity contribute to aortic aneurysm, stroke, and the development of *moyamoya* and thoracic aortic diseases^41^. *ACTA2* mutations are associated with an increased risk of acute aortic dissection^42^. ACTA2 plays an important role in the regulation of signalling processes that control myofibroblast contraction, including ERK1/2 phosphorylation during wound healing^43^; this molecule is also expected to contribute to the I/R injury healing.

ROI analysis based on MDM of MALDI-IMS data could be a powerful tool for *in situ* screening of infarction biomarkers, such as sarcomeric proteins, in cardiac tissues, and might prove useful in AMI studies. Our study was limited by the small number of tissues and the data were not strongly conclusive of ischaemic changes in cardiac tissue. More clinical studies will be required to confirm our data and the identified peptides.

## Methods

This study and all protocols used were approved by the Medical Ethics Committee of the Graduate School and Faculty of Medicine, Kyoto University, Japan. Informed consent was obtained and documented from the patient’s family. All experiments and image data analyses were performed in accordance with the relevant guidelines and regulations, including Ethical Guidelines for clinical study by Ministry of Health, Labour, and Welfare and Ministry of Education, Culture, Sports, Science, and Technology.

### Subjects

Myocardial infarction was diagnosed based on the clinical and autopsy findings, as described in the text. Three cardiac tissues were selected from a patient who was chosen from a pool of 15 subjects. Two cardiac samples included acute infarcted tissue showing contraction bands in the endocardium (samples 1 and 2) and one sample that represented intact (healthy) tissue (sample 3).

### Tissue preparation

Samples were fixed with 10% (v/v) formaldehyde in phosphate buffer (pH 7.2), and the free wall of each LV was embedded in paraffin following fixation. Paraffin-embedded blocks were sequentially cut into 4-μm sections for microscopic observation and 5-μm sections for LC/MS analysis. For diagnosis, 4-μm specimens were stained with H&E and PTAH.

### Pre-treatment procedure

FFPE tissues were cut into 3–4 μm thick sections and mounted on indium tin oxide-coated glass slides at Sigma–Aldrich (St. Louis, MO). Plates were dried overnight in a desiccator at 55 °C, and the sections were confirmed to have adhered firmly to the ITO glass.

Tissue sections on the glass slides were treated with 800 μL of the pre-treatment buffer (0.1 M NH_4_HCO_3_ and 30% (v/v) CH_3_CN), and then covered by a cover slip that was fixed by a paper bond on the glass slide to create a chamber for the incubation of cardiac tissues (i.e. pre-treatment in Fig. 1). The glass slides were incubated for 1 h at 37 °C. Each chamber was filled with 200 μL fresh pre-treatment buffer and covered with a coverslip in an air-tight manner. Slides were heated at 94 °C for 5 min on an aluminium hot plate (Dako, Glostrup, Denmark)^13^.

Following this step, 100 μL of 0.1 μg/μL trypsin (Sequencing Grade Modified Trypsin; Promega, Madison, WI) solution containing 2.5 mM NH_4_HCO_3_ and 10% (v/v) CH_3_CN was added to the chambers, and then was covered with a coverslip for 3–5 min to allow the buffer to infiltrate into the sample. Thereafter, the trypsin solution was discarded, and the sample was dried for several minutes. The slide was then incubated at 37 °C for 6 h, and after the removal of trypsin solution the sample was dried at room temperature.

### Matrix deposition

Three sample slides were placed in a slot on a MALDI target plate and affixed with conductive tape. Next, 2,5-dihydroxybenzoic acid (50 mg/mL) in a solution of 50% methanol and 0.05% trifluoroacetic acid was added to the sections using a CHIP-1000 chemical inkjet printer (Shimadzu, Kyoto, Japan). The matrix was added as 5-nL droplets by micro-spotting 25 cycles of 200 pL per spot at a spatial interval of 250 μm. After spotting, the target plate was dried in a desiccator at 20 °C.

### MS imaging and MDM

Mass spectra were acquired using a highly flexible research grade MALDI TOF/TOF mass spectrometer (AXIMA Performance and 7090 series; Shimadzu, Kyoto, Japan) equipped with a 337-nm pulsed nitrogen laser operated at a repetition rate of 10 Hz. Spectra were recorded in positive ion mode over the *m/z* range of 700 to 3000. External calibration was performed using a mixed solution of angiotensin II and adrenocorticotropic hormone fragment 18–39.

For visualization, raw MALDI-IMS data were converted into the Imaging MS Solution ver. 1.20 (Shimadzu). The structure of the MDM is shown in Fig. 1. First, we measured the intensity values for *m*/*z* = *a* of each pixel in the image and arranged the value as a column vector. Similarly, another value *m*/*z* = *b* was measured and the value was arranged as the next column vector. Further measurements of all detected intensity values *of m*/*z* (*c. d,…*) were arranged in the row of the MDM. Row and the column row vectors yielded an MDM for a single whole dataset of a tissue.

### ROI analysis

The infarction ROI annotations on the pathology image were made by a pathologist and the corresponding infarction ROI on the *m/z* image were manually mapped from pathology annotations. Intensity values of all pixels in the ROI (infarction area) were measured in the *m/z* image of each peptide. In the ROI analysis, *P-*values indicated the probability of no difference in intensity, with respect to the null hypothesis, using Student *t-*tests. Cohen’s *d-*value is defined by:

1 $$d=\frac{{s}_{{\rm{i}}}-{s}_{{\rm{h}}}}{\sqrt{\frac{({n}_{{\rm{i}}}-1){\sigma }_{i}^{2}+({n}_{h}-1){\sigma }_{h}^{2}}{{n}_{{\rm{i}}}+{n}_{h}-2}}}$$

Here, *s*_*i*_ and *s*_*h*_ represent the average signal intensity of the pixels in the infarction area (ROI) and the healthy area, respectively. The values *n*_*i*_ and *n*_*h*_ represent the pixel numbers in the infarction area (ROI) and the healthy area, respectively. The values *σ*_*i*_ and *σ*_*h*_ represent the standard deviation of the intensity in the pixels in the infarction area (ROI) and the healthy area, respectively^22^. Cohen’s criteria are as follows: *d* < 0.2: not significant, 0.2 < *d* < 0.5: small, 0.5 < *d* < 0.8: medium, and *d* > 0.8: great (significant). According to the above criteria, the *d-*values of MYH7 and ACTA2 signal intensities satisfies *d* > 0.8, an*d* there were significant differences in the intensity.

### Tandem MS and statistical analysis

MS/MS data were collected using a MALDI-QIT-TOF MS (AXIMA Resonance and MALDI-7090^TM^; Shimadzu). Spectra were exported to the Mascot search engine (Matrix Science, Boston, MA) using the following search parameters: taxonomy = *Homo sapiens*; database = SwissProt; MS tolerance = 0.2 Da; MS/MS tolerance = 0.3 Da; enzyme = trypsin; missed cleavage = 1. Peptides and proteins were identified using the Paragon algorithm provided with ProteinPilot 4.5 Beta (AB SCIEX, Danaher Corporation, Washington, D.C.) combined with the UniProt-Swiss-Prot database (version 2010-6, Homo sapiens) and sequences of the known contaminants (AB SCIEX). Matches were assigned with a significance threshold of *P* < 0.05^13^. We performed false discovery rate (FDR) analysis after using the Proteomic System Performance Evaluation Pipeline (PSPEP) software (Danaher) for peptide identification. We performed label-free quantification of peptides using Protein Quantitation 1.0 MicroApp (PQMA), which is part of Peak View 1.1.1 (Danaher). We analysed liquid chromatography-tandem mass spectrometry (LC-MS/MS) data sets for all samples using Protein Pilot 4.5 beta. Subsequently, to construct a list of peaks for all detected unique peptides, the file was imported to the Peak View 1.1.1 platform using PQMA. Protein abundance was obtained using Marker View 1.2.1. Peptides with a confidence >0.95 were selected for export. Shared peptides, including contaminating peptides, were not exported. Proteins identified based on only one peptide were excluded. Each value was normalized to the sum of the total area of individual sample. The reliability of the identification was validated by repeating the post-separation software analysis.

### Protein extraction and LC/MS

Cardiac proteins were analysed using our previously described method^16^. Briefly, samples were homogenized and suspended in 20 μL of 0.1 M NH_4_HCO_3_ that contained 30% (v/v) CH_3_CN, and then centrifuged at 10,000 × *g* for 1 min. Samples were incubated at 95 °C for 90 min, centrifuged at 10,000 × *g* for 1 min, and cooled on ice. Next, 1 μL of 1 μg/μL trypsin solution was added. Samples were incubated at 37 °C overnight. The next day, 10 mM DTT was added and the digests were heated at 95 °C for 5 min, dried, and re-suspended in 0.1% TFA containing 2% CH_3_CN to obtain a final protein concentration of 0.2 μg/μL. Samples were separated using a nano-flow reverse-phase LC (NanoLC-Ultra System; Eksigent, Dublin, CA). An aliquot of each sample (5 μL) was injected into a trap column and washed for 10 min using 0.1% formic acid. Peptides were eluted for further analyses using a quadrupole time-of-flight hybrid mass spectrometer (Triple TOF 5600 system; AB SCIEX, Framingham, MA), equipped with a nano-electrospray ionization source (NanoSpray; AB SCIEX, Framingham, MA). Tandem MS (MS/MS) scans were performed using a collision energy of 35 kV with unit-resolution.

### Immunohistochemistry

Tissue sections on the glass slides were incubated for 1 h at room temperature in 1:20 and 1:100 dilutions of anti-ATP5A and anti-MYH6 primary monoclonal antibodies, respectively (Proteintech, Manchester, UK). HRP/DAB staining (EnVision universal kit; Dako), including anti-rabbit secondary antibody incubation, was carried out according to the manufacturer’s instructions. Slides were counterstained with haematoxylin.

### Data availability

The datasets generated during and/or analysed during the current study are available from the corresponding author on reasonable request.

## Electronic supplementary material

Supplementary text, figures, and tables
