# Supplementary material for: Region of Interest analysis using mass spectrometry imaging of mitochondrial and sarcomeric proteins in acute cardiac infarction tissue
Source: Sci Rep. 2018 May 10;8:7493. doi: 10.1038/s41598-018-25817-7 (PMC5945593; doi:10.1038/s41598-018-25817-7)
Supplement: Supplementary file 1 — Supplementary text, figures, and tables [file 41598_2018_25817_MOESM1_ESM.doc]

**Supplementary Material**

**Region of Interest Analysis using mass spectrometry imaging of mitochondrial and sarcomeric proteins in acute cardiac**

**infarction tissue**

Yuka Yajima^1^*, Takuya Hiratsuka^2^§*, Yu Kakimoto^3^*, Shuichiro Ogawa^4^, Keisuke Shima^5^, Yuzo Yamazaki^5^, Kenichi Yoshikawa^6^, Keiji Tamaki^7^, Tatsuaki Tsuruyama^2^§*

*^1^ Department of Microbiology, Muroran Institute of Technology, Muroran, Hokkaido, 050-8585, Japan*

*^2^Department of Drug and Discovery Medicine, Pathology Division, Kyoto University Graduate School of Medicine, Kyoto, 606-8501, Japan*

*^3^Department of Forensic Medicine, Graduate School of Medicine, Tokai University school of Medicine, Isehara-Shimokasuya 143, Kanagawa, Kanagawa, 259-1193, Japan*

*^4^Center for Anatomical, Pathological, and Forensic Medical Research, Kyoto University Graduate School of Medicine, Kyoto, 606-8501, Japan*

*^5^Kyoto Applications Development Center, Analytical & Measuring Instruments Division, Shimadzu Corporation, 1 Nishino-kyo-Kuwabara-cho, Kyoto 604-8511, Japan*

*^6^Department of Life and Medical Sciences, Doshisha University, 1-3 Tatara Miyakodani, Kyotanabe-shi, Kyoto 610-0394, Japan*

*^7^ Department of Forensic Medicine, Kyoto University Graduate School of Medicine, Kyoto, 606-8501, Japan*

* These authors contributed equally to this study.

§ Corresponding author: Tatsuaki Tsuruyama, MD, PhD; Department of Drug and Discovery Medicine, Kyoto University, Graduate School of Medicine, Kyoto, Japan; Center for Anatomical, Pathology, and Forensic Medical Research, Kyoto University Graduate School of Medicine, Sakyoku-yohida-konoecho, Kyoto city, 606-8501, Japan

Tel.: +81 75 753 4427; Fax: +81 75 753 4493

E-mail address: [tsuruyam@kuhp.kyoto-u.ac.jp](mailto:tsuruyam@kuhp.kyoto-u.ac.jp)

and

Takuya Hiratsuka MD, PhD; Department of Drug and Discovery Medicine, Kyoto University, Graduate School of Medicine, Kyoto, Japan Sakyoku-yohida-konoecho, Kyoto city, 606-8501, Japan

Tel.: +81 75 753 4427; Fax: +81 75 753 4493

E-mail address: hiratsuka.takuya.7v@kyoto-u.ac.jp

**Supplementary Figures**

**Supplementary Figure 1.**

The whole MS spectrum of IMS of samples (*a*)1, (*b*) 2, and (*c*)3 that range from *m/z* =1000 to 2000 in Figure 3.


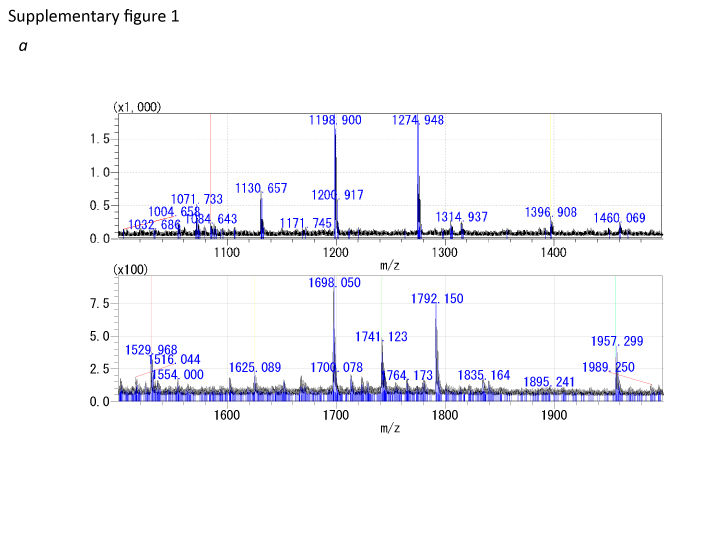


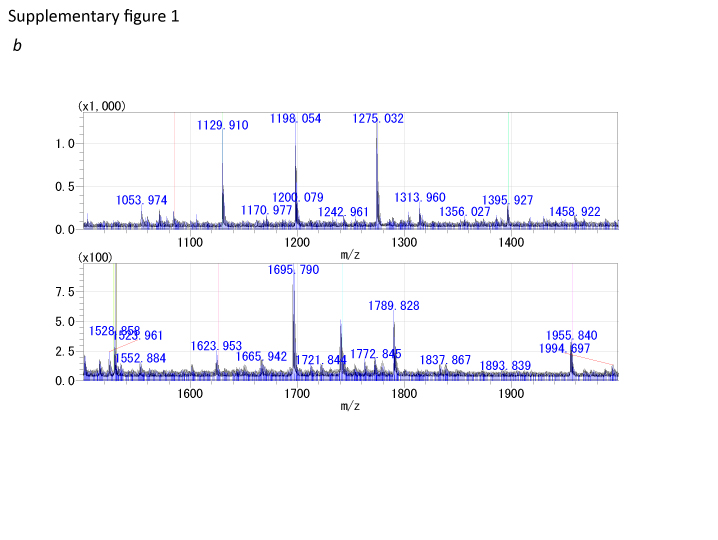


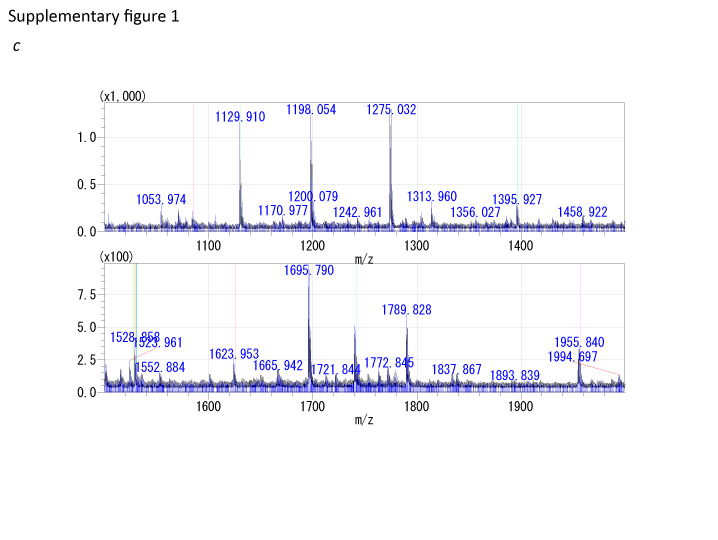


**Supplementary Figure 2.**

Tandem MS (MS/MS). Tandem mass spectrum of the precursor ion in the infarcted cardiac tissue (sample 1) at (c) MYL3, *m/z* 1396, *P* = 1.2 × 10^-2^.


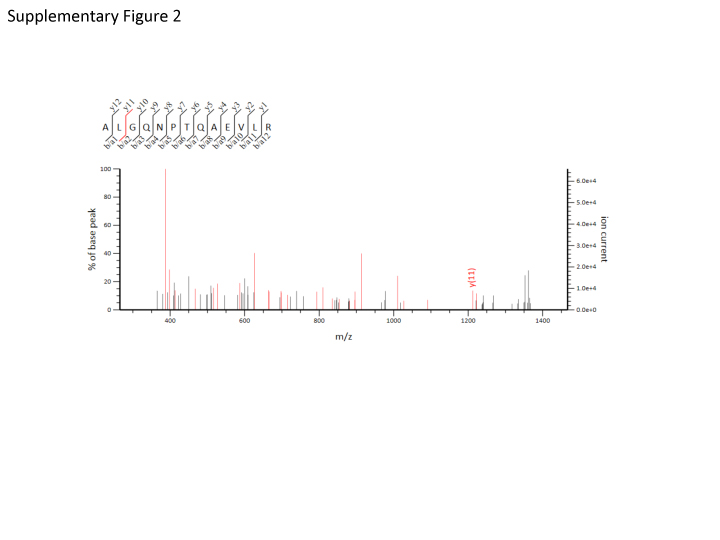


**Supplementary Figure 3. ROI analysis for *t*-test and *d*-value analysis. Left, endocardium; right, pericardium. The lines margin the measurement area.**


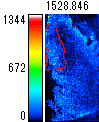

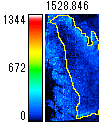


**Supplementary Table 1. Identified proteins using IMS profiling of the cardiac tissue samples.** Sample numbers indicate the patient from whom the samples were derived. MALDI-MS type indicates the IMS apparatus used in the experiment.

| *m/z* | Protein name | Expected  (*P* > 0.05) | Sample No. | MALDI-MS type |
| --- | --- | --- | --- | --- |
| 1625.0 | ATP synthase subunit alpha, mitochondrial | 6.20E-05 | No. 1 | AXIMA Resonance |
| 1624.9 | ATP synthase subunit alpha, mitochondrial | 7.20E-03 | No. 2 | AXIMA Resonance |
| 1624.9 | ATP synthase subunit alpha, mitochondrial | 9.40E-06 | No. 3 | AXIMA Resonance |
| 976.4 | Actin, aortic smooth muscle alpha 2 | 2.30E-03 | No. 1 | AXIMA Resonance |
| 976.5 | Actin, aortic smooth muscle alpha 2 | 2.10E-02 | No. 2 | AXIMA Resonance |
| 1130.6 | Actin, aortic smooth muscle alpha 2 | 2.20E-02 | No. 2 | AXIMA Resonance |
| 1130.6 | Actin, aortic smooth muscle alpha 2 | 6.40E-03 | No. 3 | AXIMA Resonance |
| 1791.0 | Actin, aortic smooth muscle alpha 2 | 1.40E-05 | No. 2 | AXIMA Resonance |
| 1791.0 | Actin, aortic smooth muscle alpha 2 | 4.10E-07 | No. 3 | AXIMA Resonance |
| 1956.1 | Actin, aortic smooth muscle alpha 2 | 3.40E-06 | No. 1 | AXIMA Resonance |
| 1956.1 | Actin, aortic smooth muscle alpha 2 | 3.90E-07 | No. 2 | AXIMA Resonance |
| 1956.1 | Actin, aortic smooth muscle alpha 2 | 1.20E-07 | No. 3 | AXIMA Resonance |
| 2228.2 | Actin, aortic smooth muscle alpha 2 | 1.80E-02 | No. 2 | AXIMA Resonance |
| 2228.2 | Actin, aortic smooth muscle alpha 2 | 5.50E-08 | No. 3 | AXIMA Resonance |
| 1515.8 | Putative beta-actin-like protein 3 | 1.10E-04 | No. 1 | AXIMA Resonance |
| 1515.8 | Putative beta-actin-like protein 3 | 1.40E-03 | No. 2 | AXIMA Resonance |
| 1515.8 | Putative beta-actin-like protein 3 | 9.30E-04 | No. 3 | AXIMA Resonance |
| 1084.6 | Myosin-6 | 1.00E-02 | No. 1 | AXIMA Resonance |
| 1084.5 | Myosin-6 | 5.50E-03 | No. 2 | AXIMA Resonance |
| 1839.0 | Myosin-6 | 1.50E-08 | No. 2 | AXIMA Resonance |
| 1839.0 | Myosin-6 | 1.30E-08 | No. 3 | AXIMA Resonance |
| 1356.8 | Myosin-7 | 3.80E-03 | No. 2 | AXIMA Resonance |
| 1535.9 | Myosin-7 | 1.40E-02 | No. 2 | AXIMA Resonance |
| 1741.0 | Myosin-7 | 2.70E-03 | No. 1 | AXIMA Resonance |
| 1741.0 | Myosin-7 | 3.60E-03 | No. 3 | AXIMA Resonance |
| 1488.8 | Myosin-1 | 1.70E-04 | No. 3 | AXIMA Resonance |
| 1396.8 | Myosin light chain 3 | 1.20E-02 | No. 1 | AXIMA Resonance |
| 1396.8 | Myosin light chain 3 | 4.60E-02 | No. 2 | AXIMA Resonance |
| 1396.8 | Myosin light chain 3 | 6.90E-03 | No. 3 | AXIMA Resonance |
| 1501.7 | Myosin light chain 3 | 1.20E-04 | No. 2 | AXIMA Resonance |
| 1071.6 | Haemoglobin subunit alpha | 4.20E-03 | No. 1 | AXIMA Resonance |
| 1529.8 | Haemoglobin subunit alpha | 2.60E-05 | No. 1 | AXIMA Resonance |
| 1529.8 | Haemoglobin subunit alpha | 1.20E-06 | No. 2 | AXIMA Resonance |
| 1529.8 | Haemoglobin subunit alpha | 1.70E-07 | No. 3 | AXIMA Resonance |
| 1834.0 | Haemoglobin subunit alpha | 7.40E-04 | No. 1 | AXIMA Resonance |
| 1833.9 | Haemoglobin subunit alpha | 3.20E-05 | No. 2 | AXIMA Resonance |
| 932.5 | Haemoglobin subunit beta | 2.70E-04 | No. 1 | AXIMA Resonance |
| 1274.8 | Haemoglobin subunit beta | 2.40E-02 | No. 1 | AXIMA Resonance |
| 1274.7 | Haemoglobin subunit beta | 1.70E-02 | No. 2 | AXIMA Resonance |
| 1314.7 | Haemoglobin subunit beta | 4.10E-03 | No. 2 | AXIMA Resonance |
| 1314.7 | Haemoglobin subunit beta | 8.30E-03 | No. 3 | AXIMA Resonance |
| 2059.1 | Haemoglobin subunit beta | 8.80E-09 | No. 3 | AXIMA Resonance |

**Supplementary Table 2. LC/MS profiling of the cardiac tissue samples.** The proteins selected for this study satisfy the following conditions: total identified peptide coverage > 30% of the amino acid sequence of the whole protein, and > 20 identified peptide fragments observed.

| N | Unused | Total | Accession | Name | Peptides  (95%) |
| --- | --- | --- | --- | --- | --- |
| 2 | 356.65 | 356.65 | sp|P12883|MYH7_HUMAN | Myosin-7 OS=Homo sapiens GN=MYH7 PE=1 SV=5 | 352 |
| 96 | 8.56 | 217.62 | sp|P13533|MYH6_HUMAN | Myosin-6 OS=Homo sapiens GN=MYH6 PE=1 SV=4 | 217 |
| 1 | 479.33 | 479.33 | sp|Q8WZ42-8|TITIN_HUMAN | Isoform Cardiac novex-1 of Titin OS=Homo sapiens GN=TTN | 215 |
| 3 | 85.48 | 85.48 | sp|P68032|ACTC_HUMAN | Actin, alpha cardiac muscle 1 OS=Homo sapiens GN=ACTC1 PE=1 SV=1 | 92 |
| 116 | 6 | 81.07 | sp|P68133|ACTS_HUMAN | Actin, alpha skeletal muscle OS=Homo sapiens GN=ACTA1 PE=1 SV=1 | 87 |
| 101 | 8.01 | 44.5 | sp|P60709|ACTB_HUMAN | Actin, cytoplasmic 1 OS=Homo sapiens GN=ACTB PE=1 SV=1 | 50 |
| 4 | 82.39 | 82.39 | sp|P35609|ACTN2_HUMAN | Alpha-actinin-2 OS=Homo sapiens GN=ACTN2 PE=1 SV=1 | 50 |
| 5 | 80.64 | 80.64 | sp|Q14896|MYPC3_HUMAN | Myosin-binding protein C, cardiac-type OS=Homo sapiens GN=MYBPC3 PE=1 SV=3 | 47 |
| 9 | 48.58 | 48.58 | sp|P02452|CO1A1_HUMAN | Collagen alpha-1(I) chain OS=Homo sapiens GN=COL1A1 PE=1 SV=5 | 41 |
| 18 | 36.88 | 36.96 | sp|P08590|MYL3_HUMAN | Myosin light chain 3 OS=Homo sapiens GN=MYL3 PE=1 SV=3 | 39 |
| 16 | 37.86 | 37.86 | sp|P02461|CO3A1_HUMAN | Collagen alpha-1(III) chain OS=Homo sapiens GN=COL3A1 PE=1 SV=4 | 34 |
| 17 | 36.96 | 36.96 | sp|P09493|TPM1_HUMAN | Tropomyosin alpha-1 chain OS=Homo sapiens GN=TPM1 PE=1 SV=2 | 34 |
| 14 | 41.36 | 41.36 | sp|P06576|ATPB_HUMAN | ATP synthase subunit beta, mitochondrial OS=Homo sapiens GN=ATP5B PE=1 SV=3 | 33 |
| 7 | 53.68 | 53.68 | sp|P17661|DESM_HUMAN | Desmin OS=Homo sapiens GN=DES PE=1 SV=3 | 32 |
| 6 | 58.26 | 58.26 | sp|Q14315|FLNC_HUMAN | Filamin-C OS=Homo sapiens GN=FLNC PE=1 SV=3 | 32 |
| 8 | 52.78 | 52.78 | sp|P02768|ALBU_HUMAN | Serum albumin OS=Homo sapiens GN=ALB PE=1 SV=2 | 31 |
| 21 | 27.68 | 27.68 | sp|P68871|HBB_HUMAN | Hemoglobin subunit beta OS=Homo sapiens GN=HBB PE=1 SV=2 | 30 |
| 11 | 44.45 | 44.45 | sp|P25705|ATP5A_HUMAN | ATP synthase subunit 5alpha, mitochondrial OS=Homo sapiens GN=ATP5A1 PE=1 SV=1 | 27 |
| 30 | 23.96 | 23.96 | sp|P08123|CO1A2_HUMAN | Collagen alpha-2(I) chain OS=Homo sapiens GN=COL1A2 PE=1 SV=7 | 26 |
| 27 | 26.22 | 26.22 | sp|P69905|HBA_HUMAN | Hemoglobin subunit alpha OS=Homo sapiens GN=HBA1 PE=1 SV=2 | 26 |
| 10 | 45.52 | 45.52 | sp|Q99798|ACON_HUMAN | Aconitate hydratase, mitochondrial OS=Homo sapiens GN=ACO2 PE=1 SV=2 | 23 |
| 13 | 42.06 | 42.23 | sp|P04264|K2C1_HUMAN | Keratin, type II cytoskeletal 1 OS=Homo sapiens GN=KRT1 PE=1 SV=6 | 23 |
| 208 | 2 | 28.77 | sp|P09493-6|TPM1_HUMAN | Isoform TPM1kappa of Tropomyosin alpha-1 chain OS=Homo sapiens GN=TPM1 | 22 |
| 22 | 27.46 | 27.46 | sp|P10916|MLRV_HUMAN | Myosin regulatory light chain 2, ventricular/cardiac muscle isoform OS=Homo sapiens GN=MYL2 PE=1 SV=3 | 22 |
| 12 | 43.86 | 43.86 | sp|P12111|CO6A3_HUMAN | Collagen alpha-3(VI) chain OS=Homo sapiens GN=COL6A3 PE=1 SV=4 | 21 |
|  |  |  |  |  |  |

**Supplementary Table 3. ROI analysis of infarction and health areas. SD, standard deviation.**

|  | Endocardium  (B) | SD | Pericardium  (C) | SD | *p* value | *d* value | estimation |
| --- | --- | --- | --- | --- | --- | --- | --- |
| 1528.846 | 115.1 | 92.5 | 90.3 | 78.4 | 2.53E-27 | 0.3 | small |
| 1624.891 | 76.8 | 61.3 | 57.5 | 45.5 | 3.57E-16 | 0.38 | small |
| 1741.836 | 113.3 | 91.9 | 65.4 | 52.5 | 3.61E-142 | 0.72 | medium |
| 1396.952 | 84.4 | 67 | 80.1 | 62.2 | 1.25E-02 | 0.07 | small |
| 1956.793 | 91.1 | 76 | 60.9 | 50.5 | 5.03E-75 | 0.51 | medium |
